# Supplementary material for: Factors associated with lenvatinib adherence in thyroid cancer and hepatocellular carcinoma
Source: PLoS One. 2023 Nov 16;18(11):e0294320. doi: 10.1371/journal.pone.0294320 (PMC10653419; doi:10.1371/journal.pone.0294320)
Supplement: Attachment — Submitted filename: ResponseToReviewers (1).docx [file pone.0294320.s001.docx]

Point-by-point response to the Editor’s and Reviewers’ comments: PONE-D-23-00635

To: Editor

We would like to thank you for your advice on our manuscript. We have revised the manuscript according to the Reviewers’ comments. Please note that we have responded to all reasonable suggestions from the Reviewers.

To: Reviewer #1

Major Comment 1:

#1 Lack of stratification and adjusted analysis

Authors performed adherence analysis together with different dosages for different carcinomas. If this is an accurate adherence study, it is necessary to add the data stratified with carcinoma types and treatment line, and to show criteria for dose reduction and withdrawal. If these factors related to the dose setting, the analysis adjusted for confounding factors is necessary.

**Response 1:**

Thank you for pointing this out. As you noted, it was necessary to add the data stratified by carcinoma type and treatment line and to show the criteria for dose reduction and withdrawal.

First, we described the stratification by cancer type and treatment line in the Results section according to your suggestion (page 8, line 145).

“In thyroid cancer, 47 (85.5%) received first-line treatment, 6 (10.9%) second-line treatment, and 2 (3.6%) third-line treatment. In hepatocellular carcinoma, 35 (74.5%) received first-line treatment, 4 (8.5%) second-line treatment, 5 (10.6%) third-line treatment, and 3 (6.4%) fourth-line treatment.”

We revised the criteria for dose reduction and withdrawal in the Methods section according to your suggestion (page 5, line 93).

“Describe the criteria for dose reduction and withdrawal. In case of hypertension Grade 2, administer antihypertensive drugs; in case of hypertension Grade 3 despite antihypertensive treatment, stop the drug and reduce the dose when resuming it. In the case of Grade 4 hypertension, the drug should be withdrawn. In case of hematologic toxicity and proteinuria Grade 3, the drug should be withdrawn until the patient recovers to the state before the start of treatment or Grade 2 or less. When resuming administration, do not reduce the dose at the first occurrence of adverse reactions, but reduce the dose at the second and subsequent occurrences of adverse reactions. If other Grade 2 or Grade 3 adverse reactions that are not tolerated are observed, the drug should be withdrawn until the patient recovers to the state before starting or to Grade 1 or below. If Grade 4 adverse reactions occur (in the case of non-life-threatening laboratory test abnormalities, the same treatment as for Grade 3 adverse reactions), administration should be discontinued.”

#2 Definition of adherence rate and discussion

The definitions of adherence rate and RDI need to be clarified. In particular, the definition of adherence rate was unclear.

For example, if RDI with 8mg /5days per a week (weekend rest dosage) is different from 4mg/a day. How would authors indicate adherence rate in these cases?

In conclusion, authors described that hypertension was a major reason for non-adherence, followed by hand-foot skin reactions and diarrhea.

While adherence is usually related to subjective, the prescribed dose is influenced by not only subjective but also objective such as proteinuria, liver injury, and so on. Adherence may be easier to maintain if the dose is decreasing. How do authors discuss about this point?

**Response 2:**

I think you are right that adherence improves when RDI decreases. Therefore, we calculated the RDI, as well as adherence, and confirmed that the RDI was similar compared to previous studies. By doing so, it was shown that the lenvatinib adherence rate and the factors associated with decreased adherence obtained in this study are data obtained after treatment with RDI was maintained to some extent. Therefore, the adherence rate and RDI were not calculated together, but separately. The definition of the adherence rate is provided on page 6, line 105. “The lenvatinib adherence rate in a cycle was defined as the number of times a patient took lenvatinib in one 28-day cycle divided by the prescribed 28 doses. The first dose was taken on day 1, then days 1-28 comprised the 1st cycle, days 29-56 the 2nd cycle, and days 57-84 the 3rd cycle.”

We have added the following in the Discussion section according to your suggestion (page 14, line 247).

“Although the RDI in this study is lower than that in previous studies, this is due to the difference between clinical trials and actual clinical practice, and it cannot be considered that the RDI is extremely low.”

#3 Carcinoma types

In clinical practice, lenvatinib has been approved for radioiodine-refractory thyroid cancer, unresectable hepatocellular carcinoma, advanced endometrial cancer, advanced renal cell carcinomas, and advanced or metastatic thymic carcinoma.

Why did authors focus on thyroid cancer and hepatocellular carcinoma? The reason why it is not a single cancer should also be stated.

**Response 3:**

This is a valid point. Lenvatinib was covered by health insurance in Japan for thyroid cancer, hepatocellular carcinoma, thymic carcinoma, endometrial cancer, and renal cell carcinoma, in that order. Patients with thyroid cancer and hepatocellular carcinoma were the subjects in the present study because it was conducted at a time when lenvatinib was commonly used for thyroid cancer and hepatocellular carcinoma. In addition, since the standard doses for thyroid cancer (24 mg/day) and hepatocellular carcinoma (12 mg for 60 kg or more, 8 mg for 60 kg or less) differed, we investigated the differences between these two cancer types.

As suggested, I added the following to the Introduction (page 4, line 72).

“Lenvatinib was covered by health insurance in Japan for thyroid cancer, hepatocellular carcinoma, thymic carcinoma, endometrial cancer, and renal cell carcinoma, in that order. In this study, patients with thyroid cancer and hepatocellular carcinoma were the subjects because in this study was conducted at a time when thyroid cancer and hepatocellular carcinoma were commonly used. In addition, since the standard doses for thyroid cancer and hepatocellular carcinoma are different, 24 mg/day, 12 mg (60 kg or more) and 8 mg (60 kg or less), we investigated the differences between these two cancer types.”

#4 Unclear strength of this study

It was difficult to understand the strength and clinical application of this manuscript, and it is necessary to show originality in this study.

It would be a better paper if it could be discussed scientifically based on results of this study rather than speculation.

For example, authors described that “In this study, pill counting, an objective evaluation method, and self-reporting, a subjective evaluation method, were combined” in discussion. Authors may consider comparing these methods to show their usefulness.

Otherwise, authors described that “The RDI reflects the physician's decision to withdraw or reduce the dose of lenvatinib, but adherence is important for oral anticancer drugs to ensure that patients take their medication at home”. Authors may investigate how outcomes changes with RDI or adherence evaluations.

**Response 4:**

Regarding the adherence evaluation, the WHO guideline (Adherence to long-term therapies: evidence for action. World Health Organization, 2003.) states that it is appropriate not only to use a single evaluation method, but also to use a combination of subjective and objective methods. One of the strengths of this study is that adherence was evaluated by appropriately combining two methods, a subjective method and an objective method, according to the WHO guidelines.

I think you are right regarding “Authors may investigate how outcomes changes with RDI or adherence evaluations.” This study clarified the rate of adherence to lenvatinib and the reasons for the decrease in adherence, but did not examine how it relates to the therapeutic effect.

With these considerations in mind, I added the following to the Discussion section (page 13, line 233) (page 18, line 337).

“and it has been suggested that medication adherence should be measured in two ways [21-22]”

References 22. Adherence to long-term therapies: evidence for action. World Health Organization, 2003.

I also added the following to the discussion section (page 15, line 268).

“Thirdly, we have not investigated the extent to which the decrease in adherence to lenvatinib and the decrease in RDI affect the treatment effect, and this is a future issue.”

Minor Comment 1:

Figure legend and table should be shown separated from the main text for easier reading.

It is difficult to distinguish where the main text continued if figure legend and table are included in the main text.

**Response 1:**

The Figure captions and tables were placed in the text of the manuscript immediately after the first quoted paragraph.

To: Reviewer #2

1. Page 7 lines 128-12o About the therapeutic lines. Do you refer to both cases (thyroid and hepatocellular carcinoma') Could you split these information? Maybe you could add how many patients in first in third and further for hepatocellular carcinoma and how many in thyroid cancer in the table. Lenva for Hepatocellular carcinoma should be in first line.

**Response 1:**

Thank you for pointing this out. Treatment lines for each carcinoma have been added in the table.

Table 1. Characteristics of the study population

| Treatment Line |  |
| --- | --- |
| Tyroid cancer |  |
| 1 / 2 / 3 / 4 | 47 (85.5) / 6 (10.9) / 2 (3.6) / 0 (0.0) |
| Hepatocellular carcinoma |  |
| 1 / 2 / 3 / 4 | 35 (74.5) / 4 (8.5) / 5 (10.6) / 3 (6.4) |

I added these things to the Result section (page 8, line 145).

In thyroid cancer, 47 (85.5%) received first-line treatment, 6 (10.9%) second-line treatment, and 2 (3.6%) third-line treatment. In hepatocellular carcinoma, 35 (74.5%) received first-line treatment, 4 (8.5%) second-line treatment, 5 (10.6%) third-line treatment, and 3 (6.4%) fourth-line treatment.

2. If we look to the therapeutic line, do you find any difference in the adherence according this point?

**Response 2:**

This is a very important point. Since in many cases, it was used as the first treatment for both thyroid cancer and hepatocellular carcinoma, there was no difference in adherence between treatment lines. We added this to the Results section (page 10, line 162).

“For thyroid cancer, the median adherence rate was 90.1% (n=55) in cycle 1, 91.4% (n=42) in cycle 2, and 96.1% (n=34) in cycle 3. In hepatocellular carcinoma, the median adherence rate was 94.9% (n=47) in cycle 1, 97.5% (n=31) in cycle 2, and 97.1% (n=26) in cycle 3.”

We added the following to the Discussion section according to your suggestion (page 15, line 253).

“In addition, there was no difference in adherence rates between treatment lines.”

3. About hypertension, do you provide any "prophylaxis" for acute adverse events?

Response 3:

Our explanation about the prevention of hypertension was inadequate. Because hypertension does not occur in everyone, no patient received prophylactic drugs for hypertension. I added the following to the Methods section (page 5, line 93).

“No prophylactic drugs for hypertension were administered.”

4. I would like to ask if you have also outcome results in order to understand if there are some differences in the outcome according to therapeutic adherence! I think that this could add a great value to the paper

Response 4:

We also think that outcomes are very important. However, in this study, we focused on clarifying the rate of adherence to lenvatinib in thyroid cancer and hepatocellular carcinoma, as well as the factors that reduce adherence. The lack of examining the relationship between adherence and treatment outcomes will be added as a limitation.

I added the following to the Discussion section (page 15, line 268).

“Thirdly, we have not investigated the extent to which the decrease in adherence to lenvatinib and the decrease in RDI affect the treatment effect, and this is a future issue.”
